# Identification of KRAS G12V associated clonal neoantigens and immune microenvironment in long-term survival of pancreatic adenocarcinoma

**DOI:** 10.1007/s00262-021-03012-4

**Published:** 2021-07-13

**Authors:** Chao Wang, Min Shi, Lei Zhang, Jun Ji, Ruyan Xie, Chao Wu, Xianchao Guo, Ying Yang, Wei Zhou, Chenhong Peng, Henghui Zhang, Fei Yuan, Jun Zhang

**Affiliations:** 1 Department of Oncology, Ruijin Hospital, Shanghai Jiao Tong University School of Medicine, No. 197 Ruijin er Road, Shanghai, 200025 China; 2 Department of Pathology, Ruijin Hospital, Shanghai Jiao Tong University School of Medicine, No. 197 Ruijin er Road, Shanghai, 200025 China; 3 Genecast Biotechnology Co., Ltd, Wuxi City, 214104 Jiangsu China; 4 Shanghai Institute of Digestive Surgery, Ruijin Hospital, Shanghai Jiao Tong University School of Medicine, No. 197 Ruijin er Road, Shanghai, 200025 China; 5 VIP Health Center, Ruijin Hospital, Shanghai Jiao Tong University School of Medicine, No. 197 Ruijin er Road, Shanghai, 200025 China; 6 Department of Surgery, Ruijin Hospital, Shanghai Jiao Tong University School of Medicine, No. 197 Ruijin er Road, Shanghai, 200025 China; 7 State Key Laboratory of Oncogenes and Related Genes, Shanghai Jiao Tong University, Shanghai, 200032 China

**Keywords:** KRAS G12V, Neoantigen, Pancreatic adenocarcinoma, Immune

## Abstract

**Objective:**

To investigate the molecular characteristics in tumor immune microenvironment that affect long-term survival of patients with pancreatic adenocarcinoma (PAAD).

**Methods:**

The tumor related genetic features of a female PAAD patient (over 13-year survival) who suffered from multiple recurrences and metastases, and six operations over one decade were investigated deeply. Genomic features and immune microenvironment signatures of her primary lesion as well as six metastatic tumors at different time-points were characterized.

**Results:**

High-frequency clonal neoantigenic mutations identified in these specimens revealed the significant associations between clonal neoantigens with her prognosis after each surgery. Meanwhile, the TCGA and ICGC databases were employed to analyse the function of KRAS G12V in pancreatic cancer.

**Conclusions:**

The genomic analysis of clonal neoantigens combined with tumor immune microenvironment could promote the understandings of personalized prognostic evaluation and the stratification of resected PAAD individuals with better outcome.

**Supplementary Information:**

The online version contains supplementary material available at 10.1007/s00262-021-03012-4.

## Introduction

Pancreatic ductal adenocarcinoma (PDAC) is one of the most notorious solid malignancies with the median overall survival (OS) less than 20 months after radical operation [1, 2]. However, a minority of PDAC patients survived more than 5 years. The factors affecting long-term survivors (LTSs) of PDAC are still poorly understood. Paniccia et al. reported the survival data of 431 long-term survivors with over 10-year survival among 11,081 PDAC patients underwent radical pancreatectomy, indicating that the involvement status of lymph nodes, adjuvant chemotherapy, as well as the pathologic T stage acted as the predictive factors for LTSs [3]. In addition, the possibility of long-term survival in PDAC patients could not be excluded simply according to the positive nodes or the tumor size, which implied some unknown factors simultaneously affected LTSs beyond our knowledge.

It was reported that several alterations of 4 common PDAC driver genes (*KRAS*, *TP53*, *CDKN2A* and *SMAD4*) were associated with poor outcomes of resected PDAC patients [4], while these alterations seem to be inapplicable in PDAC LTSs. Sequencing analyses of 35 resected PDAC patients survived over 10 years revealed that no significant difference in somatic alterations between LTSs and control arms, suggested that the mutation status of common driver genes might not be the determinant of PDAC LTSs[5].

Regards the tumor expansion, the immune microenvironment, certain infiltrating immune cells (i.e., CD3, CD4, CD8, CD68 positive cells) were thought to play certain roles in progression-free survival (PFS) of PDAC after radical resection [6], yet it is need to be further verified in PDAC LTSs. Angelova et al. characterized the spatiotemporal interplay between tumor-intrinsic characteristics and extrinsic immune selection based on 11-year follow-up of two metastatic colorectal cancer patients [7]. The tumor cells harboring alterations with low immunogenicity were observed to escape attacks by the immune system and to spread than forms metastases. Whereas, the tumor clones with high immunogenicity were cleared under immune selection pressure, namely immunoediting [7]. Nonetheless, little is known about the evolutionary dynamics considering the immuno-oncology (I-O) for PDAC. To date, there are few studies focusing on the integration of genomic features, immune microenvironment signatures as well as dynamics of tumor evolution, to interpret the reason of extremely low OS of PDAC patients. Additionally, rare case report of long-term survivors of PDAC make it difficult to reveal the underlying mechanisms.

Herein, we enrolled a rare case of PDAC patient with over 13 years survival, who first conducted pancreatectomy in 2007 and experienced multiple recurrences and metastases along with six operations over one decade. Comprehensive analyses of her primary and metastatic lesions were performed by using whole exome sequencing (WES), RNA-sequencing and multiplex immunohistochemistry (mIHC). The differences in molecular characteristics of this patient versus other resected PDAC patients with either long or short OS were also compared. Besides, in view of the perspective that PDAC LTSs can be identified by high-quality neoantigens [8], in silico prediction of neoantigens based on WES data was performed in the present study as well. Our results revealed that the exceptional long-term survival of this patient could be partially explained by her unique persistent neoantigens which retained during the whole disease progression. Furthermore, certain characteristics of immune microenvironment identified in this patient were supported by survival analysis of pancreatic adenocarcinoma patients in TCGA-PAAD cohort.

Therefore, we believe that the combinational effect of multi-dimensional factors mentioned above might be responsible for this extraordinary long-term survivor with PDAC following pancreatectomy.

## Materials and methods

### Patients and samples

All tumor samples of the 8 resected PDAC patients enrolled in this study and their clinical data were collected from Ruijin Hospital in Shanghai, China. This study was approved by Medical Ethical Committee of Shanghai Ruijin Hospital and performed in accordance to relevant guidelines and regulations.

### Whole exome sequencing

Exome sequencing and variants analysis of tumor specimens were performed at a commercial CAP-certified laboratory (GeneCast Biotechnology Co., Beijing). DNA from primary carcinoma and metastatic tissue specimens was extracted by using blackPREP FFPE (formalin-fixed paraffin-embedded) DNA Kit (Analytik Jena, GER) according to the manufacturer’s instructions. Extracted DNA was quantified by Qubit dsDNA HS Assay kit (Life Technologies, California, USA). Then DNA was fragmented into 150–200 bp by using Covaris M220 Focused-ultrasonicator™ Instrument (Covaris, Massachusetts, USA), and respective libraries were captured by Roche NimbleGen SepCap EZ MedExome kit. The captured DNA fragments were subjected to a NovaSeq 6000 system (Illumina) for paired end sequencing with a mean coverage of 100 × NovaSeq Control Software was used for sequencing data collection. After trimming adaptor sequences, obtained reads were subsequently mapped to the reference human genome (hg19) with BWA-MEM (v0.7.12). The duplicated reads were removed by using NovoSort (3.08.00), and local realignment around InDels (insertions and deletions) along with base recalibration was performed by using GATK (v3.7). SNV calling was performed using GATK (v4.0.7.0, Mutect2) [9] and annotated via Oncotator [10] and ANNOVAR [11]. Somatic SNVs were filtered out through the following criterions: A. SNVs with Vaf ≥ 1% and depth ≥ 30X; B. SNVs without strand bias; C. SNVs in the exonic regions; D. SNVs with corresponding allele frequency ≤ 0.002 in both the Exome Aggregation Consortum (ExAC) database[12] and Genome Aggregation Database (gnomAD). CNV was analyzed by CNVkit (v0.9.2) [13] using paired normal sample, and the cutoff of CNV gain is copy number ≥ 3, and the cutoff of CNV loss is copy number ≤ 1.2.

### Neoantigen prediction

Somatic mutant peptides with immunogenicity, namely neoantigens, were predicted by calculating their binding affinities to patients’ HLA alleles. Novel 9-11 AA (amino acid) peptides from all somatic mutations identified in each sample were determined. Then, the binding affinities of these mutated peptides or wild-type peptides to each patient’s HLA alleles were calculated by using netMHC-4.0 [14]. HLA typing for selected PDAC patients in this study was performed by using HLA-HD algorithm [15]. Those novel peptides that had a binding affinity with the half-maximal inhibitory concentration below 500 nm or 50 nm and the affinity ratio of wild-type peptides to mutant peptides over 1.5 were predicted as neoantigens or strong neoantigens, respectively [8, 16]. Neoantigenic clonal fraction (NCF) was calculated as the mean frequencies of 4 neoantigenic trunk mutations (KRAS p.G12V, TFR2 p.R771H, TNR p.V577I, ZBTB5 p.V41M) in primary lesion and 6 metastatic tumors of Pt204.

### Multiplex immunohistochemistry

Immunostaining, imaging and quantification of simultaneous detection for multiplex molecules within the tumor immune microenvironment in FFPE tumor slides were performed as previously described [17]. Two staining panels for mIHC were used in this work. Panel 1 was for PanCK, CD3, CD8 staining, and panel 2 was for CD4, CD68, CD163 staining. Firstly, 4-μm-thickness slides containing carcinoma tissue were deparaffinized in xylene, then rehydrated, and finally washed in tap water before epitope retrieval by microwave treatment in Tris–EDTA (pH 9.0). Then, the slides were preincubated with antibody diluent/block (72,424,205; PerkinElmer, Massachusetts, USA) for blocking endogenous peroxidase and followed by incubation with certain primary antibody. Labeling of above markers was performed one by one, namely incubation of primary antibody, incubation of secondary antibody, TSA visualization, followed by new round of labeling next marker. Primary antibodies used in this study were as follows (clone name, catalog number, dilution ratio): PanCK (AE1/AE3, ZM0069, 1:100), CD3 (LN10, ZM-0417, 1:100), CD8 (SP16, ZA0508, 1:100), CD4 (UMAB64, ZM0418, 1:100), CD68 (KP1, ZM0060, 1:500), CD163 (10D6, ZM0428, 1:200), all these primary antibodies were purchased from ZSGB-BIO, Beijing, China. Primary antibody of CD4 was incubated overnight at 4 ℃, and other primary antibodies were incubated for 1 h at room temperature. Subsequently, sample incubation with Opal Ploymer HRP Ms + Rb (2,414,515; PerkinElmer, Massachusetts, USA) was performed at 37 ℃ for 10 min. TSA visualization was performed by using Opal seven-color IHC Kit (NEL797B001KT; PerkinElmer, Massachusetts, USA). The stained slides were scanned by PerkinElmer Vectra (Vectra 3.0.5; PerkinElmer, Massachusetts, USA). Quantitative analyses were performed by using InForm Advanced Image Analysis software (inForm 2.3.0; PerkinElmer, Massachusetts, USA) that was trained for specific algorithms regarding tissue segmentation, cell segmentation, phenotyping tool and positivity score through pre-test with the same type of carcinoma tissue sample. before formal experiment of the examined sample. Generally, more than 15 fields per slide were included to calculate the number, percentage and density of positive cells in all nucleated cells under the 200 × magnification, and an average percentage of specific cell population was used for further analysis.

### RNA-sequencing

Expression level of Immunology-Oncology (I-O) related genes, markers or pathways were measured by Oncomine™ Immune Response Research Assay (Cat. No. A32881, Thermo Fisher Scientific, USA), an RNA-based NGS assay targeting 395 genes was conducted as previously described [18]. Briefly, RNA was extracted from FFPE tumor specimens by using the truXTRAC FFPE extraction kit (Covaris, USA). Isolated RNA was quantified by using Qubit™ RNA HS Assay Kit (Thermo Fisher Scientific, USA) and then reversely transcribed into cDNA. RNA libraries were prepared by amplifying with pooled primers targeting 395 genes and sequenced on the Ion PGMTM Systems according to the manufacturer’s instructions. Gene expression was initially calculated as reads per million (RPM), and normalization ratio determined by RPM of 10 housekeeping genes was used to normalize RPM counts for each gene in the examined samples. The manufacturer defines 35 I-O related functional pathways with different gene sets for data analysis. Additionally, some immune signatures including cytolytic markers, IFN-γ signature, chemokines and HLA molecules were used as reference obtained from previous studies [19, 20]. The immune signature scores were calculated by averaging the log_10_ transformed expression values of related genes in each signature.

### TCR sequencing

Oncomine™ TCR-Seq β libraries were generated with leukocyte total RNA according to the Ion AmpliSeq™ library preparation protocol. Standard kit conditions for template preparation and sequencing used the Ion 510™ Kit—Chef and the Ion S5™ Sequencing System, respectively, for evaluation of the generated libraries. All libraries were sequenced on Ion 530 Chips. All results were analyzed using the Ion Torrent Browser and the TCRβ Repertoire Plug-in.

### Data acquisition and characteristics

Mutation data, neoantigen counts, RNA-sequencing data and associated clinical data from PAAD patients and all other types of cancer in TCGA cohort were download from cBioPortal (https://www.cbioportal.org). Mutation data and associated clinical data from PAAD patients in ICGC (International Cancer Genome Consortium) cohort were downloaded from ICGC-Canada. The following cases were excluded with: [1] missing data and [2] insufficient survival information. RNA-sequencing data were used to calculate immune signature scores of certain immune cell subsets by GSEA method as previously described [21]. All processes were executed by using R software.

### Statistical analysis

Statistical analyses were performed by using R software version 4.0.2. Kaplan–Meier curves were generated to estimate OS of PAAD patients from TCGA and ICGC datasets, and Log-rank (Mantel-Cox) test was used to assess differences of OS, disease-specific survival, disease-free interval and progression-free interval between patients. *P* < 0.05 represents statistical significance.

### Data availability

All data related to this study can be found in the main text or the supplementary materials. Detailed mutation lists, neoantigen prediction results of our selected patients, TCR sequencing data as well as TCGA -PAAD and ICGC-PAAD datasets were shown in Additional Table 1, Table 2, Table 3, Table 4 and Table 5, respectively.

## Results

### Case report of a pancreatic cancer patient

**Fig. 1:**
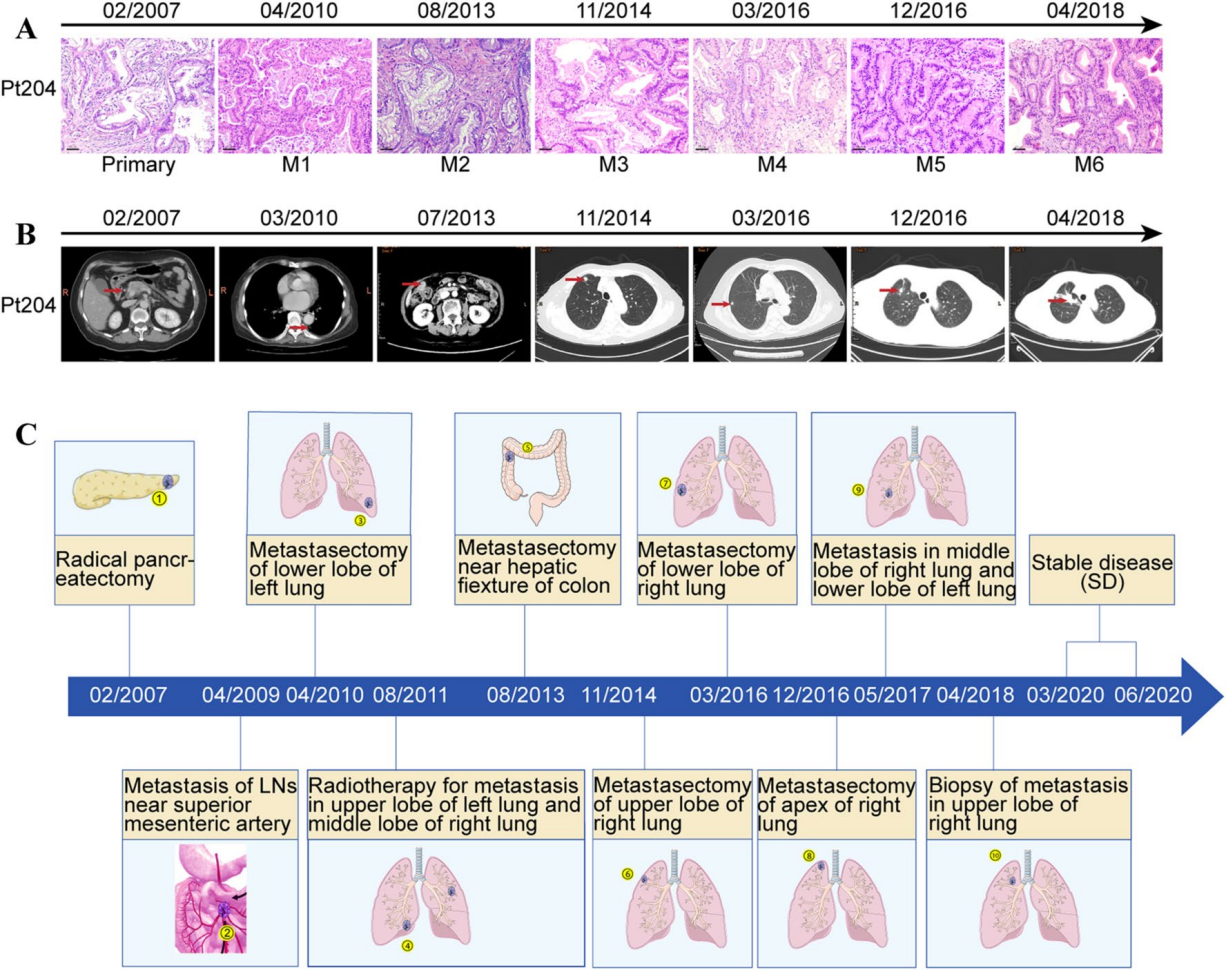
Clinical outcomes of Pt204 from 2007 to 2018. **a** Histology of Pt204’s primary and metastatic tumors. Scale bar, 50 μm, 200 × magnification. **b** Representative radiological images related to Pt204’s outcomes during metastatic progression. **c** Schematic depicting of whole treatment course of Pt204

In the case of patient Pt204, a 66-year-old female, was diagnosed with pancreatic cancer and later subjected to radical pancreatectomy and splenectomy in February 2007. Pathological examination showed a stage Ib (pT2N0M0) of moderately-differentiated PDAC invading the wall of portal vein without metastasis in all resected lymph nodes. The adjuvant chemotherapy was conducted based on a Gemcitabine regimen for the first 2 years (03/2007–01/2009). Subsequently, Pt204 received six cycles of metastectomy, chemotherapy, target therapy and radiotherapy for clearance of metastatic foci (Fig. 1a & b). To date, Pt204 is still alive with stable disease (Fig. 1c).

### Mutational profiles of Pt204 and other PDAC patients

**Fig. 2:**
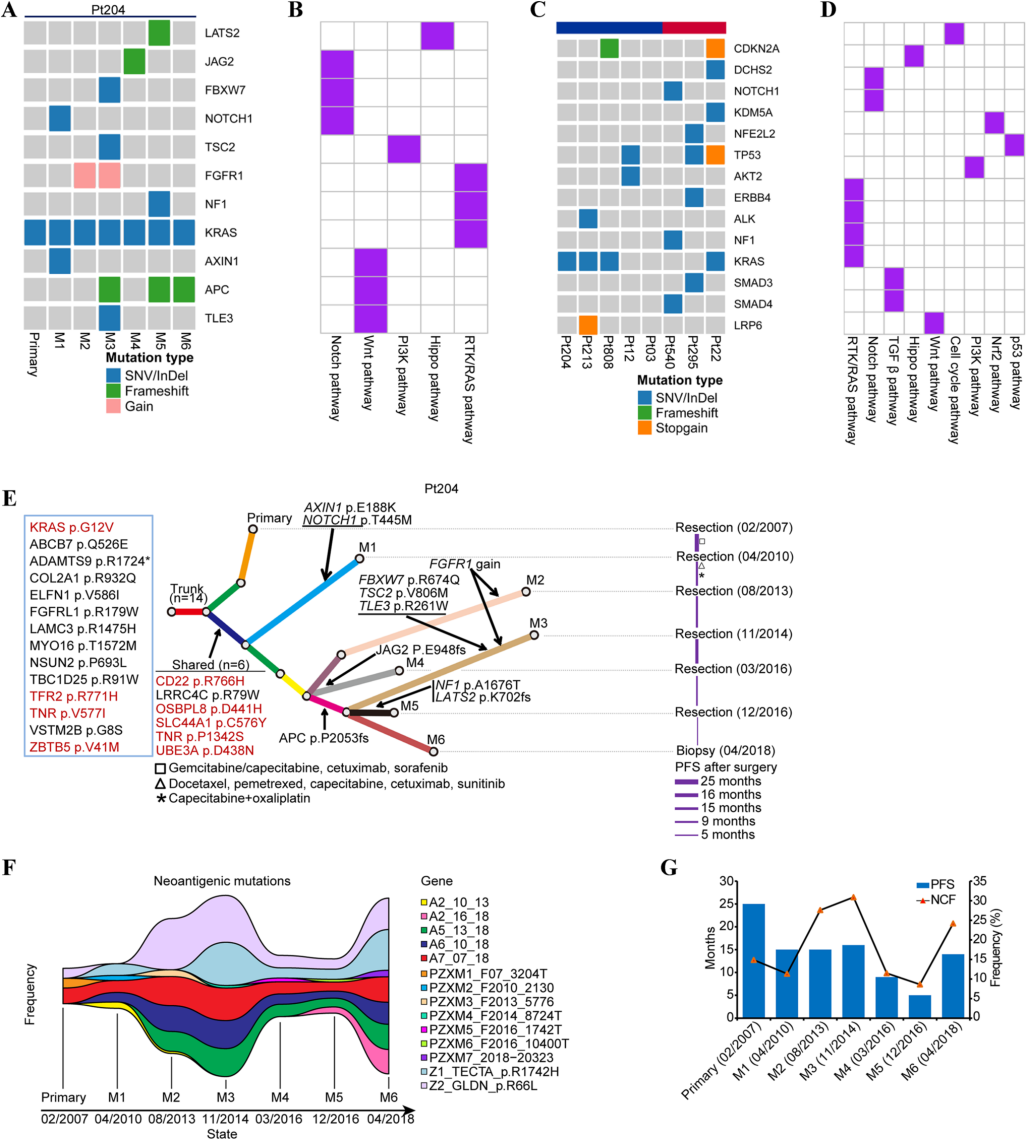
Genomic features of Pt204 and other seven PDAC patients. **a **& **b** Mutation profiles of ten curated oncogenic signaling pathways in primary foci along with metastatic foci (M1-M6) of Pt204. **c** & **d **Characterization of gene mutations of cancer-associated pathways in primary foci of Pt204 and other seven PDAC patients. **e** A phylogenetic tree of Pt204 was depicted according to Pt204’s somatic alterations in primary and metastatic foci. Highlighted alterations were trunk and shared mutations or those involved in cancer pathways. Red marked mutations were neoantigenic mutations in silico. Pt204’s progression-free survival (PFS, month) after each surgery and treatment information were displayed chronologically. F. The shift of frequencies of predicted neoantigenic mutations in seven tumor foci of Pt204 over time. Different colors denoted specific neoantigenic mutations shared in two or more tumor foci or observed in only one tumor focus. G. Similar variation trend of the length of Pt204’s progression-free survival (PFS, month) after each surgery and Pt204’s neoantigenic clonal fraction (NCF, %) calculated by the mean frequencies of four neoantigenic trunk mutations in seven samples

To explore the dynamic progression of pancreatic cancer in multiple dimensions, whole exome sequencing (WES) was performed on macrodissected tumor foci (M1, 04/2010; M2, 08/2013; M3, 11/2014; M4, 03/2016; M5, 12/2016; M6, 04/2018). According to the defined genes of 10 curated signaling pathways in TCGA [22], KRAS G12V mutation was detected in primary foci and all metastatic foci of Pt204, while other common reported driver genes in PDAC (TP53, CDKN2A, SMAD4) were not mutated in the case of Pt204 [4] (Fig. 2a&b). Besides, genetic variants of seven other PDAC patients with different prognosis were also identified by WES, as shown in Fig. 2c&d, KRAS G12V mutation was also present in primary foci of patients Pt213 (OS > 30 months), Pt808 (OS > 5 years) and Pt22 (OS > 23 months). The clinical data of these eight patients and their tumor specimens were listed in Table 1, Table 2.

**Table 1:** Clinicopathologic data of eight PDAC patients selected for survival after surgery

Patient ID	Sex	Age at surgery (y)	Tumor location	Grade	Margin status	TNM	Stage (AJCC 8th Ed.)	Post-operative therapy	Overall survival (m)
Pt204	F	66	Body	G2	R0	pT2N0M0	IB	Chemotherapy, radiotherapy, target therapy	150 +
Pt213	M	51	Body-Tail	G2	R0	pT2N1M0	IIB	Chemotherapy, radiotherapy	41 +
Pt808	M	67	Head	G2	R0	pT2N1M0	IIB	Chemotherapy, radiotherapy	110
Pt12	F	63	Body-Tail	G2	R0	pT2N1M0	IIB	Chemotherapy	95 +
Pt03	M	60	Body-Tail	G2	R0	pT2N0M0	IB	Chemotherapy	31 +
Pt540	M	55	Head	G3	R0	pT2N1M0	IIB	Chemotherapy	11
Pt295	M	55	Body-Tail	G3	R0	pT3N1M0	IIB	Chemotherapy	9
Pt22	M	63	Head	G3	R0	pT2N0M0	IB	Chemotherapy	23

**Table 2:** Clinical information of primary lesion and six metastatic tumors of Pt204

Samples of Pt204	Time-point	Age (y)	Type of surgery	Post-operative therapy	PFS (m)
Primary	02/2007	66	Resection	Chemotherapy, target therapy	25
M1	04/2010	69	Resection	Chemotherapy, target therapy	15
M2	08/2013	72	Resection	Chemotherapy	15
M3	11/2014	74	Resection	None	16
M4	03/2016	75	Resection	None	9
M5	12/2016	76	Resection	None	5
M6	04/2018	77	Puncture	Chemotherapy	14

Evolutionary landscape of mutations among primary and metastatic lesions of Pt204 was performed in Fig. 2e. Not only KRAS p.G12V but also other 13 rare point mutations with uncertain significance were detected in all Pt204’s samples. Likewise, there were 6 scarce missense mutations shared by all Pt204’s metastases but absent in the primary lesion (CD22 p.R766H, LRRC4C p.R79W, OSBPL8 p.D441H, SLC44A1 p.C576Y, TNR p.P1342S and UBE3A p.D438N). It should be noted that 4 trunk mutations (KRAS p.G12V, TFR2 p.R771H, TNR p.V577I and ZBTB5 p.V41M) and 5 common mutations (CD22 p.R766H, OSBPL8 p.D441H, SLC44A1 p.C576Y, TNR p.P1342S and UBE3A p.D438N) in metastases (red marked in Fig. 2e) of the 20 shared mutations were predicted to generate neoantigens. The frequencies of these neoantigenic mutations were generally high (maximum of 44%) and varied among different samples (Additional Table 1). In contrast to few high-frequency clonal neoantigenic mutations observed in multiple specimens of Pt204, most low-frequency neoantigenic mutations were merely present in a specific sample (Fig. 2f and Additional Table 1). A previous study reported that *MUC16* neoantigens could enrich in PDAC LTSs [8], unexpectedly, there was no *MUC16* mutation detection in any sample of Pt204. The alterations of *MUC16* were present in other patients (excluding Pt213) in our study, but none of them met the standards for predicting neoantigen generation (Additional Table 1 and Additional Table 2).

### Impact of clonal neoantigens on progression-free survival of Pt204

Whether the differential frequencies of above clonal neoantigenic mutations were associated with prognosis of Pt204, the mean frequencies of 4 neoantigenic mutations (*KRAS* p.G12V, *TFR2* p.R771H, *TNR* p.V577I, *ZBTB5* p.V41M) from the trunk in primary lesion and 6 metastatic tumors were calculated as neoantigenic clonal fraction (NCF) for Pt204’s each sample. Interestingly, the variation trend of NCF in 7 samples appeared to be in accordance with the length of PFS following each surgery (Fig. 2g), which indicated that the higher NCF of certain sample was generally followed by the longer PFS after surgery at corresponding time point and vice versa. For instance, both the NCFs (30.89%, 11.48% and 8.61%) of samples M3/M4/M5 and the PFSs [16 months, 9 months and 5 months] after three surgeries of Pt204 from Nov, 2014 to Dec, 2016 demonstrated the declining tendency, at the time point of M5, both Pt204’s NCF and post-operative PFS were the lowest values (Fig. 2g). Nevertheless, the NCF of sample M6 (04/2018) did not keep dropping and rebounding to 24.19%, which was in accordance with the prolonged PFS after Pt204’s biopsy in April 2018 compared with that of Dec, 2016 [14 months versus 5 months] (Fig. 2G).

### Immune microenvironment signature of Pt204

**Fig. 3:**
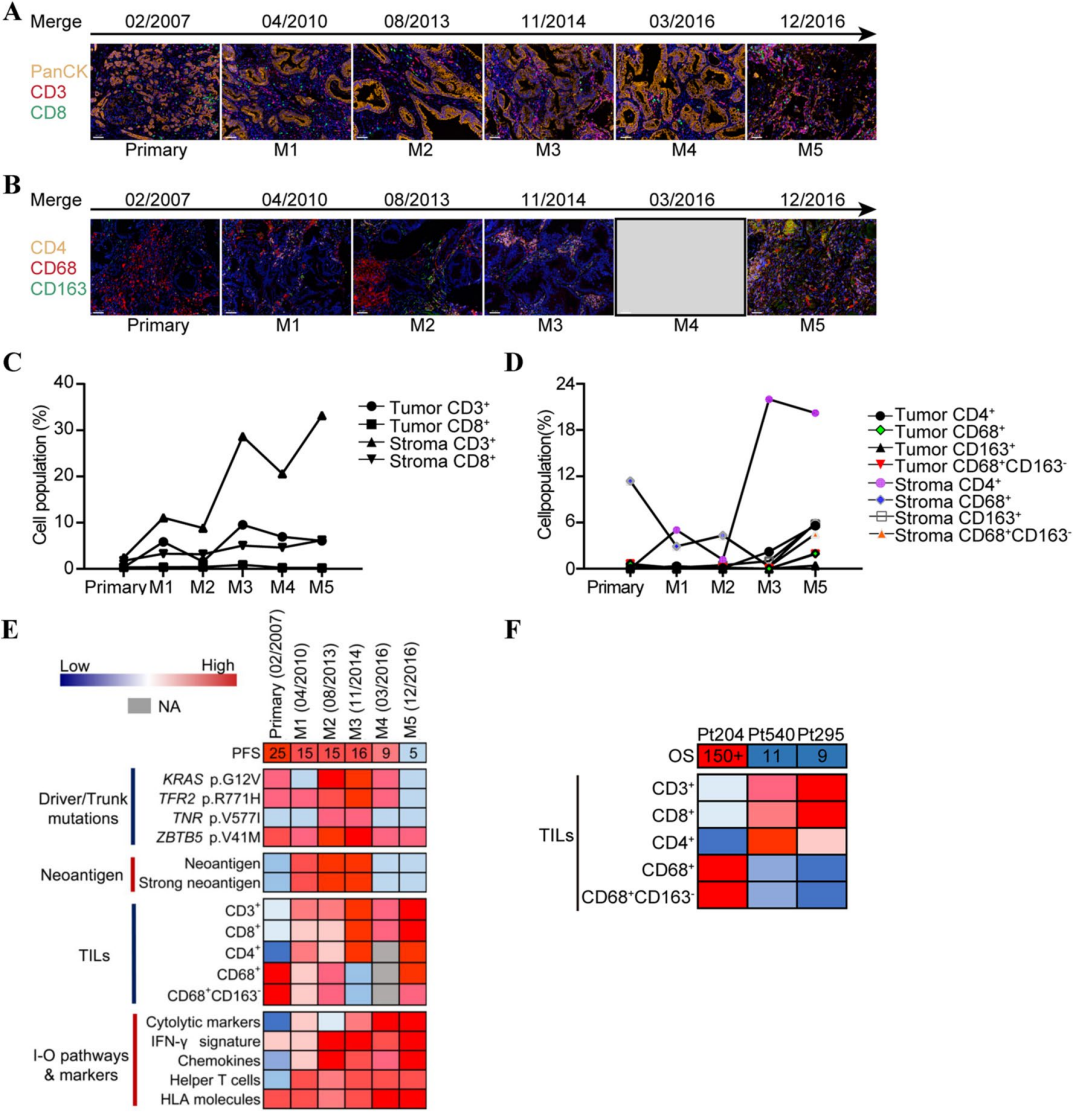
The relevance of neoantigenic mutations with altered frequencies to outcomes of Pt204 and Pt204’s tumor immune microenvironment signatures. **a** Representative mIHC images of CD3/CD8 TILs in six samples of Pt204. PanCK, pan cytokeratin. Nuclei (blue) were counter-stained by DAPI. Scale bar, 50 μm, 200 × magnification. **b** Representative mIHC images of CD4/CD68/CD163 TILs in five samples of Pt204. Nuclei (blue) were counter-stained by DAPI. Scale bar, 50 μm, 200 × magnification. **c** Quantitative mIHC results of CD3/CD8 TILs from 15 random vision fields in six samples of Pt204. **d** Quantitative mIHC results of CD4/CD68/CD163 TILs from 15 random vision fields in five samples of Pt204. **e** Comprehensive heatmap of diverse aspects potentially related to progression-free survival (PFS, month) of Pt204 after each surgery according to Pt204’s driver/trunk mutation status, neoantigen counts, TILs and gene expression of certain I-O pathways and markers in seven samples. **f** Integrated heatmap of multiple TILs expression level in primary lesion of patient Pt204 and short-term survival patients Pt540 and Pt295

Immune microenvironment signatures were examined in primary and metastatic tumors of Pt204. Briefly, there were very few CD3/CD8/CD4 positive TILs (tumor infiltrating lymphocytes) in Pt204’s primary lesion, whereas the obviously elevated degrees of infiltration of CD3, CD8 or CD4 positive lymphocytes were observed in her metastases of M1-M5 (Fig. 3a-d). Instead, the infiltration level of CD68 positive cells, which stand for macrophage[23], in stroma region of primary lesion was much higher than that in metastases (Fig. 3b&d). Whereas, CD163 was negative in most CD68^+^ macrophages. Immune signature scores of gene expression levels of immune related pathways or markers [18–20] were also calculated and incorporated together (Fig. 3e). In short, the gene expressions involved in cytolytic markers, IFN-γ signature, chemokines and helper T cells were generally higher in metastases over primary tumor of Pt204, which was basically consistent with Pt204’s infiltration profiles of CD3/CD8/CD4 positive cells. Besides, the gene expression of HLA molecules was always kept at relatively higher levels in all samples of Pt204. Above diverse results (driver mutation, neoantigen and immune microenvironment) related to the outcome of Pt204 were integrated in Fig. 3e. Furthermore, two relatively short-term survival patients (Pt540: OS:11 months and Pt295: OS: 9 months) in our cancer center were also enrolled, and slices of primary lesions were staining by multiplex immunohistochemistry assay and compared with those of patient Pt204. As shown in Figure S2 and Fig. 3f, there were obvious higher level of CD3, CD8 or CD4 positive tumor infiltrating lymphocytes in stroma region of primary lesion in Pt295 and Pt540 rather than those in Pt204, while the CD68^+^ macrophages and CD68^+^CD163^−^macrophages were enriched in stroma region of Pt204 and almost not present in those of Pt295 and Pt540.

Additionally, we performed TCR (T cell receptor) sequencing of the peripheral blood sample collected from this patient in her most recent follow-up (03/2020). The TCR CDR3 (complementarity-determining region 3) β sequences obtained from peripheral blood T cell repertoire were compared with reported neoantigen [24, 25] or tumor associated antigen-specific CDR3β sequences [26–28]. Using the Hamming Distance algorithm [29], several T cell clones were found possessing very similar CDR3β sequences to the KRAS G12V or MAGEA1 epitope-specific TCRs (Distance ≤ 1). The proportions of these clones in the peripheral T cell population were relatively higher (additional Table 3).

### KRAS^high^ was associated with adverse outcomes in pancreatic cancer

TCGA databases were utilized to investigate the mRNA expression of KRAS in different tumors and normal tissues of multiple cancer types. compared with adjacent normal tissues, the KRAS expression was significantly higher in ACC (*P* < 0.001), BRCA (*P* < 0.001), CESC (*P* < 0.001), CHOL (*P* < 0.001), COAD (*P* < 0.001), ESCA (*P* < 0.001), GBM (*P* < 0.001), HNSC (*P* < 0.01), KICH (*P* < 0.001), KIRC (*P* < 0.05), LAML (*P* < 0.001), LGG (*P* < 0.001), LIHC (*P* < 0.001), LUAD (*P* < 0.001), LUSC (*P* < 0.001), OV (*P* < 0.001), PAAD (*P* < 0.001), PRAD (*P* < 0.001), SKCM (*P* < 0.001), STAD (*P* < 0.001), TGCT (*P* < 0.001), THCA (*P* < 0.001), UCS (*P* < 0.001). However, KRAS mRNA expression was significantly lower in READ (*P* < 0.001) compared with adjacent normal tissues (Figure S1A).

Whether KRAS expression was correlated with prognosis in pancreatic cancer patients, the TCGA-PAAD cohort was chosen to evaluate the impact of KRAS expression on survival rates. The results showed that the pancreatic cancer patients belonging to KRAS^high^ expression group exhibited relatively shorter overall survival time (*P* = 0.0011, HR = 1.03, 95% CI: 1.01–1.05), shorter disease-specific survival time (*P* = 0.00045, HR = 1.04, 95% CI: 1.01–1.06), shorter disease-free interval time (*P* = 0.00089, HR = 1.06, 95% CI = 1.02–1.1) and shorter progression-free interval time (*P* = 0.0011, HR = 1.02, 95% CI = 1–1.04), compared with KRAS^low^ expression group (Figure S1B-E). Therefore, it is conceivable that high KRAS expression is an independent risk factor which leads to a poor prognosis in pancreatic cancer patients.

### Bio-informational analyses of KRAS in Pancreatic cancer

**Fig. 4:**
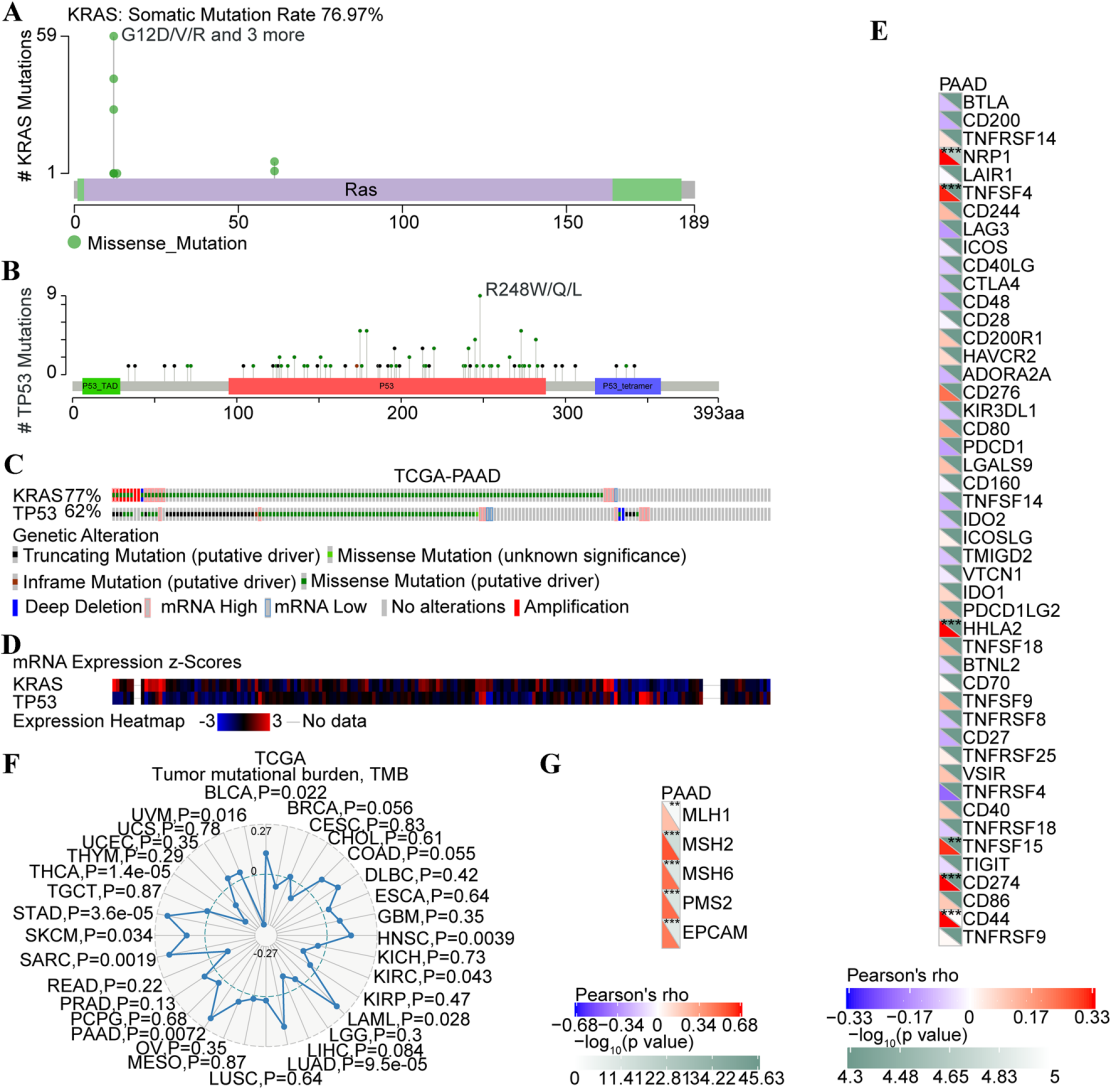
Bio-informational analyses of *KRAS* in pancreatic cancer. **a** Screenshot of *KRAS* mutation frequencies from the cBioPortal website. **b** Screenshot of *TP53* mutation frequencies from the cBioPortal website. **c** Oncoprint demonstrating the frequency of *KRAS* and *TP53* mutations in the TCGA-PAAD cohort. **d** Heatmap demonstrating the mRNA expression of *KRAS* and *TP53* in the TCGA-PAAD cohort. **e** Correlation between the expression of *KRAS* and immune checkpoint molecules in TCGA-PAAD cohort. **f** Correlation between the expression of *KRAS* and tumor mutational burden (TMB) in pan-cancer database. **g** Correlation between the expression of *KRAS* and *MMRs* genes (MLH1, MSH2, MSH6, PMS2 and EPCAM)

A previous study had mentioned that the compact association between the alterations of four main driver genes (*KRAS*, *CDKN2A*, *SMAD4* and *TP53*) and the outcome of pancreatic adenocarcinoma patients [4]. Thus, it is worth further investigating the alteration of drive genes in PDAC patients. In present study, gene alterations of *KRAS* and *TP53* in pancreatic cancer were analyzed by using cBioportal web tool. As shown in Fig. 4a, commonly occurring mutant forms of *KRAS* were G12A, G12C, G12D, G12R, G12V, G13D, Q61L and Q61H. In terms of *TP53*, mutations mainly existed in the DNA binding domain, and the one hot spot (R248W/Q/L) represent the commonly known mutations in the DNA binding domain of *TP53* (Fig. 4b). As shown in Fig. 4c, *KRAS* have been altered in 77% cases of TCGA-PAAD cohort, and *TP53* have been altered in 62% cases of TCGA-PAAD cohort, respectively. The mRNA expression of *KRAS* and *TP53* was also examined in each case of TCGA-PAAD cohort (Fig. 4d).

### Correlation analyses between *KRAS* expression and immune marker sets

To further investigate the relationship between *KRAS* and the diverse immune infiltrating cells, we analyzed the correlations between *KRAS* and immune markers sets of various immune cells in TCGA-PAAD cohort. The results found that *KRAS* mRNA expression was positively correlated with NRP1, TNFSF4, HHLA2, TNFSF15, CD272 and CD44 (Fig. 4e).

Previously reported studies mentioned that the tumor mutation burden (TMB) is a potential biomarker to predict the efficacy of immunotherapy, as well as tumor combined with high TMB might possess more neoantigens which could be recognized by a host immune system [30, 31]. Therefore, it is necessary to assess the relationship between TMB and *KRAS* expression. Our results of pan-cancer analyses found that *KRAS* mRNA expression is positively correlated with TMB in pancreatic cancer patients (*P* = 0.0072, Fig. 4f). Besides, the bulk of clinical evidence favored the use of ICIs in MSI-H solid tumor [32], and a classical hallmark feature of MSI-H colorectal cancer was a crucial lymphocytic infiltrate correlated with a higher neoantigen load [33]. Thus, there was a growing interest in gaining a better understanding of the MSI landscape in pancreatic cancer. As shown in Fig. 4g, *KRAS* mRNA expression was positively associated with MLH1 (*P* < 0.01), MSH2 (*P* < 0.001), MSH6 (*P* < 0.001), PMS2 (*P* < 0.001) and EPCAM (*P* < 0.001) (Fig. 4G).

### KRAS G12V was prognostic of survival in pancreatic cancer

**Fig. 5:**
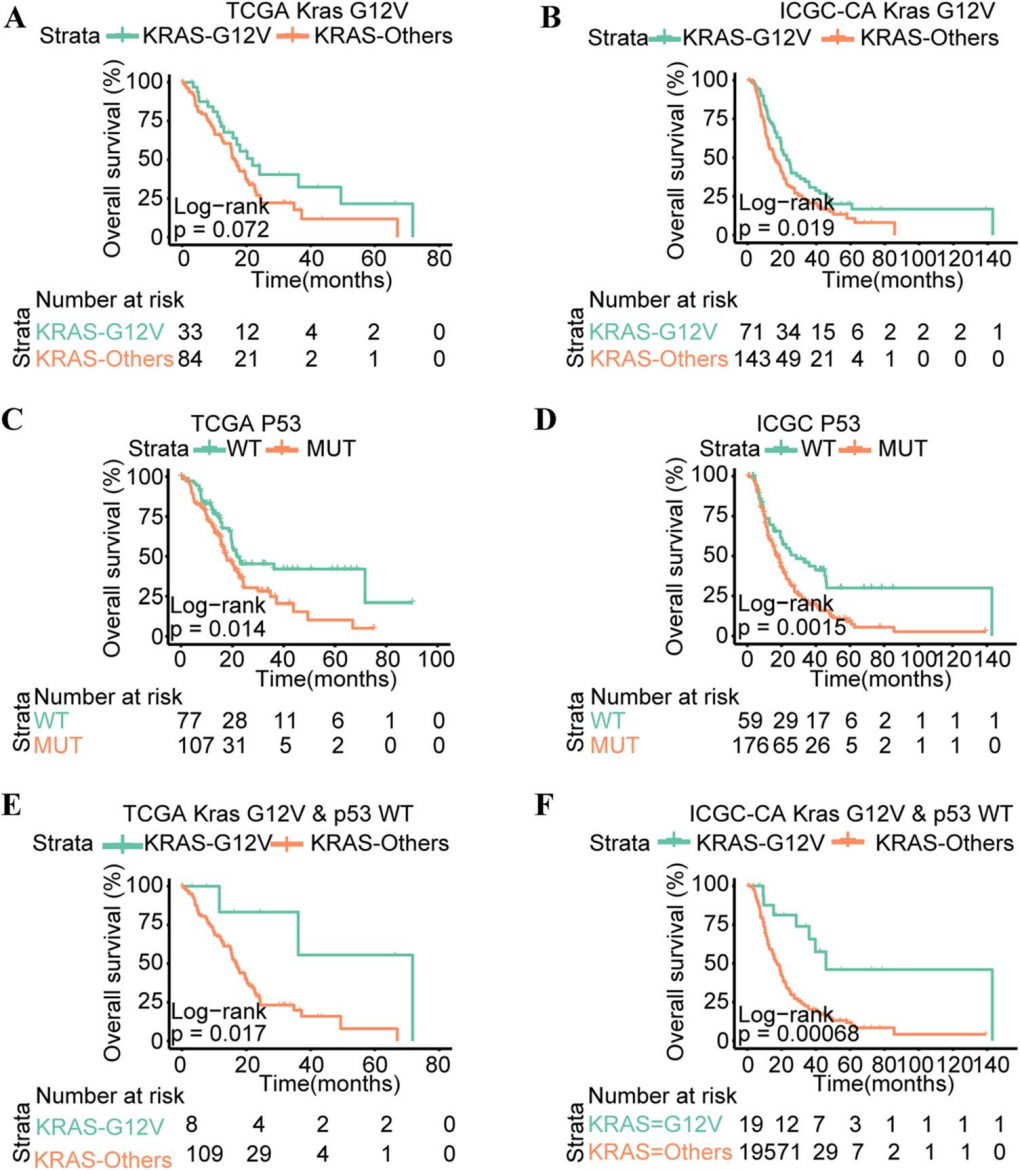
*KRAS* G12V, *TP53* wild-type and combined *KRAS* G12V and *TP53* wild-type identify long-term pancreatic cancer survivors in TCGA-PAAD and ICGC-PAAD (Canada) cohort. **a** Overall survival stratified by mutation (G12V) in KRAS in the TCGA-PAAD cohort. **b** Overall survival stratified by mutation (G12V) in *KRAS* in the ICGC-PAAD (Canada) cohort. **c** Overall survival stratified by mutation in TP53 in the TCGA-PAAD cohort. **d** Overall survival stratified by mutation in *TP53* in the ICGC-PAAD (Canada) cohort. **e** Overall survival of patients with tumors harboring *KRAS* G12V and *TP53* wild-type or others in the TCGA-PAAD cohort. **f** Overall survival of patients with tumors harboring *KRAS* G12V and *TP53* wild-type or others in the ICGC-PAAD (Canada) cohort. Horizontal bars indicate median values. Number at risk is the number of biologically independent samples in individual patients. *P* values were determined using a log-rank test

Pancreatic cancer patients with *KRAS* G12D mutation were reported with relatively poor outcomes. While compared with *KRAS* G12D mutation, *KRAS* G12V mutation has been identified as a protective factor in terms of disease-free survival and OS [4]. We also found that *KRAS* G12V mutation existed in primary foci and metastatic foci of Pt204. To further investigate the prognostic value of *KRAS* G12V in pancreatic cancer, the TCGA-PAAD and ICGC-PAAD (Canada) cohorts were utilized and results demonstrated that *KRAS*^G12V^ patients exhibited relatively longer overall survival time than patients with other mutation types of KRAS, yet the prognostic value of KRAS^G12V^ in TCGA-PAAD cohort was marginal significant (TCGA/KRAS^G12V^: *P* = 0.072, ICGC/KRAS^G12V^: *P* = 0.019; Fig. 5a&b). *TP53* wild-type (*TP53*^WT^) and mutation (*TP53*^MT^) were also examined in TCGA-PAAD and ICGC-PAAD (Canada) cohorts and results found that *TP53*^WT^ patients have obviously longer overall survival tine (TCGA/*TP53*^WT^: *P* = 0.014, ICGC/*TP53*^WT^: *P* = 0.0015; Fig. 5c&d). Combining *KRAS*^G12V^ and *TP53*^WT^ into consideration, we found that *KRAS*^G12V^ and *TP53*^WT^ patients have significantly better prognosis compared with patients with other mutation types of KRAS and *TP53*^WT^ in TCGA-PAAD and ICGC-PAAD cohorts (TCGA/*KRAS*^G12V^&*TP53*^WT^: *P* = 0.017, ICGC/*KRAS*^G12V^&*TP53*^WT^: *P* = 0.00068; Fig. 5e&f and additional Tables 4&5).

## Discussion

To our knowledge, in the setting of multiple recurrences and metastases, Pt204 may be the first reported case of PDAC individual with a survival rarely exceeded 13-year after multiple metastasectomies. Short and intermediate term improvements in survival of PDAC patients following pancreatectomy were confirmed in 15,604 resected PDAC patients, but their long-term survival (≥ 5 years) failed to be improved by pancreatic resection [34]. Hence, repeated surgeries are insufficient to account for the extraordinary survival of Pt204. In fact, most relapsed PDAC patients tend to progress aggressively and be unable to undergo next surgery. It is undeniable that Pt204’s negative lymph node, earlier stage and various post-operative therapies are indeed favorable factors for PDAC LTSs as mentioned before [3]. It seems that certain innate features other than clinicopathologic factors are likely to be the determinants for long-term survival of Pt204.

An impressive phenomenon is that Pt204 has a number of high-frequency clonal neoantigenic mutations during whole disease course. Although Pt204 received diverse drug treatments, none of these clonal neoantigenic mutations disappeared during metastatic progression from 2007 to 2018. Functionally, except for *KRAS* p.G12V, these clonal neoantigen-related mutations are not recorded or confirmed as pathogenic mutations in public database (COSMIC, OncoKB, cBioPortal, etc.). Importantly, the variation of NCF calculated from 4 neoantigenic trunk mutations in 7 samples exhibited considerable relation to Pt204’s length of PFS after each surgery. In consideration of the universal consensus that PDAC is driven by oncogenic *KRAS* combined with many cancer genes [35], it can be inferred that these rare neoantigenic mutations may work together with *KRAS* p.G12V as well as other genetic alterations in cancer-related signaling pathways to influence progression and outcomes of Pt204 in the past 13 years. Certainly, there are still some key problems worthy to be explored.

For one thing, how did neoantigens possibly produced by 9 clonal neoantigenic mutations mentioned above battle with the oncogenic alterations to maintain such long-term survival of Pt204? It is widely believed that the neoantigens derived from tumor-specific somatic mutations have the potential to be recognized by the immune system and allow specific CD8^+^ cytotoxic T cells to kill cancer cells as a consequence of activated immune response [33, 36]. Nonetheless, not all neoantigenic mutations inevitably initiate the desirable anti-tumor immune response. In fact, clonal neoantigens (high clonal fraction) generated by certain immunogenic mutations present in most cancer cells probably elicit immune rejection by T cells, whereas subclonal neoantigens (low clonal fraction) from the minority of cancer cells may fail to lead to effective immune elimination [37, 38]. Presumably, the presence of most neoantigens might be attribute to their high mutation frequency, for which drive effects caused by *KRAS* p.G12V, and other pathogenic mutations might be lowered partially under a stronger anti-tumor immunity triggered by augmented neoantigens, resulting in prolonged PFS after surgery and accompanied higher NCF at certain time-points of Pt204. Instead, with the drop of NCFs in Pt204’s metastases M3/M4/M5, the length of PFS was almost synchronously shortened. In other words, in the context of immune selection, most subclonal neoantigenic alterations with low frequencies were indeed immunoedited or cleared completely, but the high-frequency clonal mutations with immunogenicity were not eliminated and retained in subsequent metastases, which might be strongly linked to the sustained immune response during Pt204’s disease course. Thus, it is plausible that Pt204’s long-term survival can be regarded as a kind of controlled tumor growth or staged balance between oncogenic effects of driver mutations versus durable anti-tumor immunity related to clonal neoantigenic mutations.

Considering post-operative therapy received or not, her 10-year survival (from February 2007 to December 2016) after pancreatectomy can be segmented into two phases. In the former phase from February 2007 to August 2013, she underwent 3 surgical resections and corresponding post-operative or preoperative treatments including various chemotherapies and target therapies, while Pt204’s PFS after the 3 surgeries were 25, 15 and 15 months, respectively (Table 2). Nonetheless, in the latter phase until December 2016, Pt204 did not receive any systemic treatment and the PFS after the other 3 surgeries of this period were 16, 9 and 5 months, respectively (Table 2). That is to say, the systemic therapy markedly improved Pt204’s PFS after surgical resections. It should be noted that CD3^+^/CD8^+^ TILs were lack in Pt204’s primary tumor in which genes related to cytolytic markers, IFN-γ signature and chemokines were universally low expressed, whereas both infiltration levels of CD3/CD8 positive T cells and gene expression of those pro-inflammatory pathways or markers were significantly elevated in metastases after post-operative systemic therapy. Theoretically, cytotoxic chemotherapy may strengthen the anti-tumor immunity via promoting the release of antigen peptides from tumor cells and thereby resulting in immunogenic cell death or synergize with immunotherapies [39, 40]. Given this, chemotherapy plus target therapy facilitate the release of neoantigens produced by neoantigenic mutations and enhance immune response in metastatic tumors which lead to better outcomes of Pt204. Furthermore, post-operative treatments appeared to obtain the relative growth advantage of those cancer cells harboring 9 common neoantigenic mutations over other malignant cells without these mutations, which was evidenced by the elevated NCF of sample M2 (08/2013) compared with sample M1 (04/2010).

Furthermore, what is the relationship between immune microenvironment signatures and outcomes of PDAC? In contrast to the conception of the greater densities of CD3^+^/CD8^+^ T cells the longer OS or PFS in PDAC (6, 8, 41), there were very few CD3/CD8 positive T cells in the primary lesion of Pt204. In the presence of high-frequency neoantigenic mutations alone in primary lesion with lack of CD8^+^ T cell infiltrates, this patient still achieved long-term survival after pancreatectomy. Thus, we presumed that abundant CD8^+^ T cells in resected PDAC might not be essential to LTSs.

Finally, above-mentioned features possibly associated with clinical outcomes of Pt204 may not be just an exception. Owing to high malignancy and poor prognosis of PDAC, it is difficult to perform a large-scale longitudinal analysis of PDAC LTSs. In fact, such case as Pt204 with super LTS is an extremely rare research object in clinical practice of PDAC. To our best, we deeply investigated the multi-dimensional profiles of Pt204’s including 7 primary and metastatic tumors at different time-points across 10 years, and our results were partly supported by TCGA and ICGC data together with some preceding studies, implicating that certain phenomena or rules observed from Pt204 might be universally valid for other PDAC patients.

## Conclusion

In summary, high-frequency clonal neoantigenic mutations in combination with featured immune microenvironment may impact on the resected PAAD patients to obtain a long-term survival or better outcome. In view of good condition of Pt204 until now, our findings from retrospective analysis have opportunity to be prospectively tested in future follow-up, which could provide new evidence to establish a promising set of biomarkers for prognosis evaluation of resected PDAC and guide their clinical management.

### Supplementary Information

Below is the link to the electronic supplementary material.

Supplementary file1 (XLSX 211 KB)

Supplementary file2 (XLSX 73 KB)

Supplementary file3 (XLSX 14 KB)

Supplementary file4 (XLSX 39 KB)

Supplementary file5 (XLSX 65 KB)

## Data Availability

All data generated or analyzed during this study are included in this paper.

## Abbreviations

PAAD: Pancreatic adenocarcinoma
PDAC: Pancreatic ductal adenocarcinoma
OS: Overall survival
LTSs: Long-term survivors
PFS: Progression-free survival
WES: Whole exome sequencing
mIHC: Multiplex immunohistochemistry
FFPE: Formalin-fixed paraffin-embedded
AA: Amino acid
I-O: Immunology-Oncology
ICGC: International Cancer Genome Consortium
NCF: Neoantigenic clonal fraction
TILs: Tumor infiltrating lymphocytes
TCR: T cell receptor
CDR3: Complementarity-determining region 3
TMB: Tumor mutation burden
